# Protein expression of eIF4E and integrin αvβ6 in colon cancer can predict clinical significance, reveal their correlation and imply possible mechanism of interaction

**DOI:** 10.1186/2045-3701-4-23

**Published:** 2014-04-28

**Authors:** Zhengchuan Niu, Jiayong Wang, Shahbaz Muhammad, Weibo Niu, Enyu Liu, Cheng Peng, Benjia Liang, Qi Sun, Shinichi Obo, Zhaobin He, Song Liu, Xueqing Zou, Jun Niu

**Affiliations:** 1 Department of Hepatobiliary Surgery, Qilu Hospital, Shandong University, Jinan, Shandong 250012, PR China; 2 Key Laboratory of Cardiovascular Remodeling and Function Research, Chinese Ministry of Education and Public Health, Jinan, Shandong 250012, PR China

**Keywords:** eIF4E, Integrin αvβ6, Colon cancer, TMA, ERK, mTOR

## Abstract

**Background:**

Both eukaryotic translation initiation factor 4E (eIF4E) and integrin αvβ6 play an important role in the development and progression of cancer. The aim of this study was to investigate the expression of eIF4E and Integrin αvβ6, their clinical significance as well as the two proteins’ correlation in colonic carcinoma tissues.

**Results:**

The expression levels of eIF4E and integrin αvβ6 were analyzed in colon cancerous and paraneoplastic tissues of 138 cases via tissue microarray (TMA)- immunohistochemistry. And their clinical significance as well as the two proteins’ correlation was also investigated. The expression of eIF4E was significantly associated with clinical TNM stage (P = 0.009), while T stage (P = 0.011) and TNM stage (P = 0.012) were significantly associated with integrin αvβ6 expression. Moderately weak correlation exists between the two proteins (r =0.299, P <0.001). The survival analysis by Kaplan-Meier and Cox regression model showed that protein expression of high eIF4E and positive integrin αvβ6, as independent prognostic factors (RR = 2.417, P = 0.001 and RR = 2.393, P = 0.001), tended to have a significantly poorer 5-year survival rate (P = 0.013 and 0.025, respectively, the log-rank test).

**Conclusion:**

eIF4E and Integrin αvβ6 were indicators of tumor’s progression and poor prognosis of patients with colon cancer. And the potential signaling loop involving them may provide a helpful therapeutic target in prevention and treatment of colon cancer.

## Introduction

Colon cancer is one of the leading causes of cancer-related deaths worldwide. With the improvement of current living standards and environmental degradation, its morbidity has been on the rise year by year [1]. Both eIF4E and integrin αvβ6 can play important roles in the development and progression of colonic carcinoma [2-4].

Eukaryotic initiation factor 4E (eIF4E), a 25-kDa cap binding protein, delivers cellular mRNAs to the eIF4F translation initiation complex by binding the 5′-cap structure of these mRNAs [5]. Only about 10% of mRNAs with 5′UTRs of sufficient length (>200 nt) and three-dimensional steric hindrance require the assistance of the eIF4E for efficient translation [6]. eIF4E overexpression can selectively affect transport of specific transcripts and up-regulate the translation of a limited pool of mRNAs with long G-C rich 5′-UTRs and complex structure, encoding proteins involved in cellular growth, angiogenesis, survival, tumorigenesis, and metastasis,such as cyclin D1, c-myc, vascular endothelial growth factor (VEGF), fibroblast growth factor (FGF-2), matrix metalloprotease 9 (MMP-9) [2,7-9]. It is considered as a central regulator of malignancy. eIF4E overexpression has been demonstrated in carcinomas of breast, colon, prostate, bladder, cervix, lung, head and neck (reviewed in [9]).

Integrins are a family of heterodimeric cell-adhesion molecules composed of two non-covalently bound transmembrane subunits (18 α-subunits and 8 β-subunits reported) [10]. These proteins collectively mediate cellular adhesion to extracellular matrix (ECM) and modulate cell proliferation, migration, invasion, and survival by activating intracellular signaling pathways [11]. The β6 integrin subunit with αv, its sole binding partner, is an epithelial-cell-restricted antigen, which is reported to be expressed in epithelial cancers (lung, breast, pancreas, ovary, oropharynx and colon), as well as during tissue remodeling events (e.g., fibrosis, wound healing) [12,13]. Integrin αvβ6 plays an pivotal role in various aspects of cancer progression [14].

Integrin-mediated signals have been known to contribute to malignancy by regulating transcription [15]. On the other side, recent studies indicated they could also selectively enhance translation initiation of protein synthesis relating to malignancy, through activation of eIF4E via signal pathway of PI-3 K/Akt/mTOR and Ras/ERK/MNK [16-18]. However, the correlation between eIF4E and integrin αvβ6 and their possible interaction at molecular level are still not clearly known. The aim of our present study was to explore the coexpression and clinical significance of eIF4E combined with integrin αvβ6 in colon cancer, which could provide a theoretical basis for further study of molecular mechanism in therapeutic intervention.

## Results

### The expression of integrin β6 and eIF4E in normal and neoplastic colon tissue

**Figure 1:**
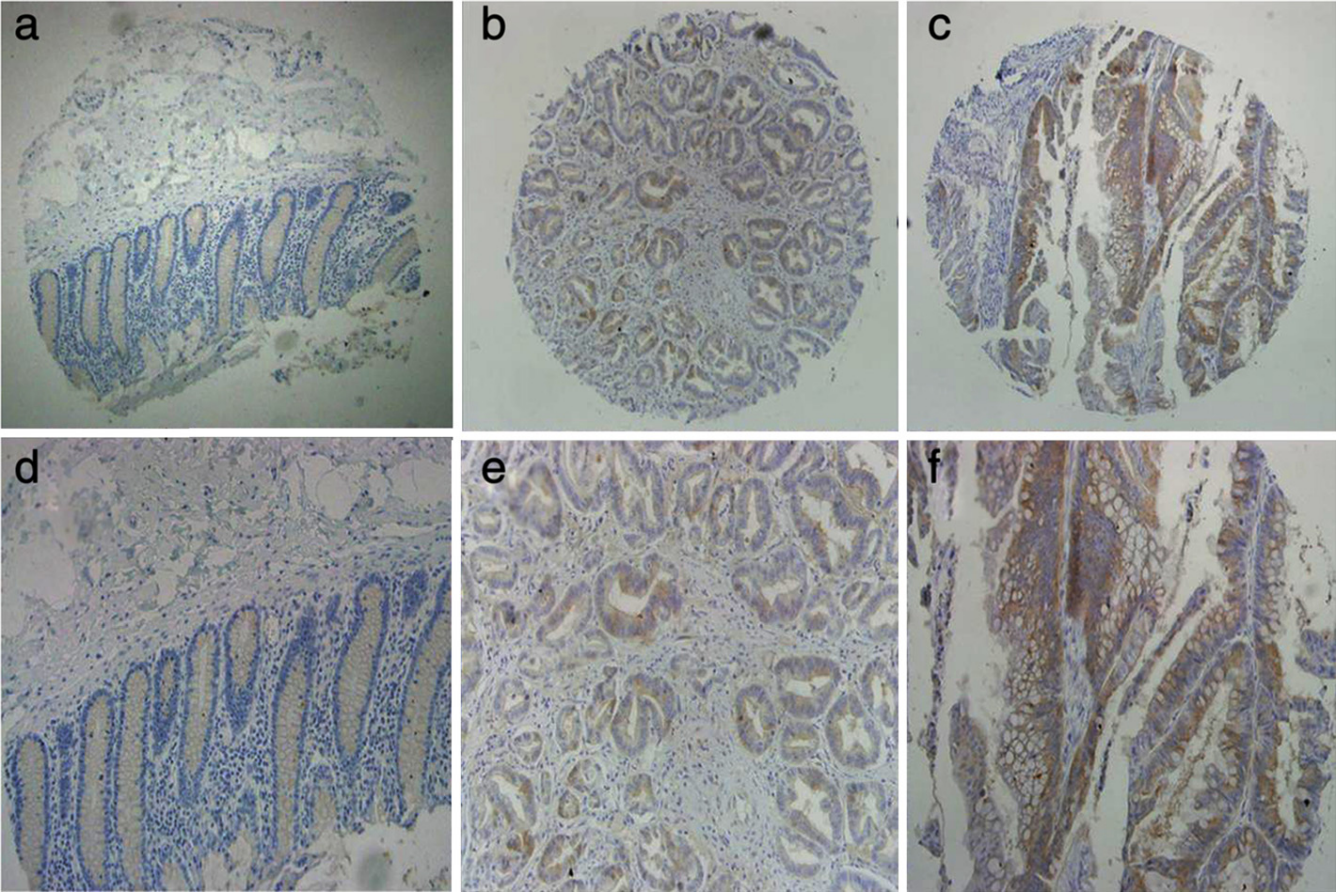
**eIF4E expression in human colonic cancer.** First row: **(a)**, **(b)**, **(c)** (Scale bar 200 μm.); second row: **(d)**, **(e)**, **(f)**, corresponding to each figure above (Scale bar 100 μm). **(a)** and **(d)** was obtained from paracancerous normal tissue of patient with colon cancer, with negative staining. **(b)** and **(e)** was obtained from colonic carcinoma tissue, with low eIF4E expression. **(c)** and **(f)** was obtained from colonic carcinoma, with high eIF4E expression.

**Figure 2:**
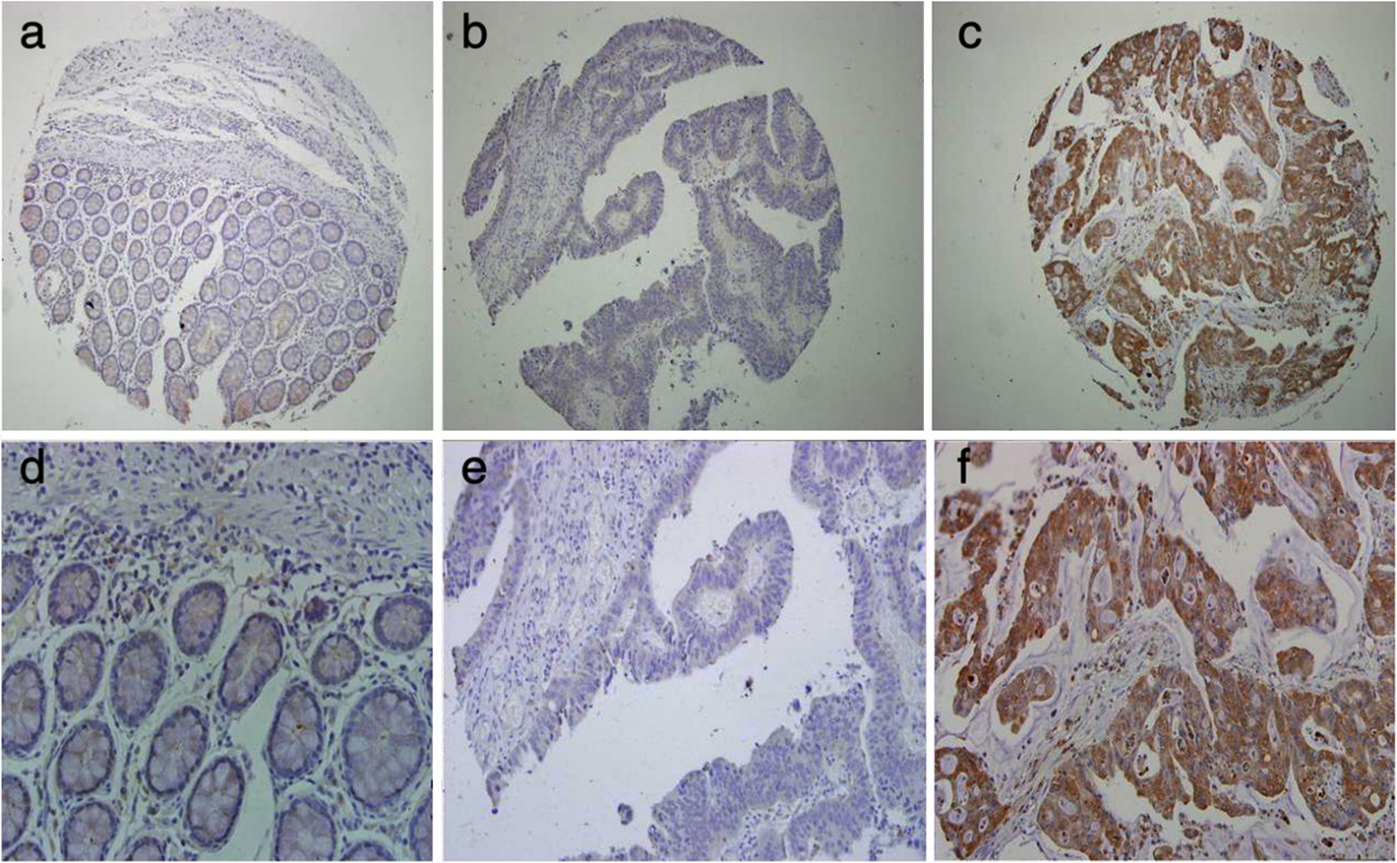
**Integrin β6 expression in human colonic cancer.** First row: **(a)**, **(b)**, **(c)** (Scale bar 200 μm.); second row: **(d)**, **(e)**, **(f)**, corresponding to each figure above (Scale bar 100 μm). **(a)** and **(d)** was obtained from paracancerous normal tissue of patient with colon cancer, with negative staining. **(b)** and **(e)** was obtained from colonic carcinoma tissue,with negative Integrin β6 expression. **(c)** and **(f)** was obtained from colonic carcinoma, with positive Integrin β6 expression.

For eIF4E cohort, 10 tumor cases could not be stained as negative expression, and positive rate was 92.8%, with 74 high expression (Figure 1, Table 1). For integrin αvβ6, 54 cases were positive expression, and 84 were negative (Figure 2, Table 1). And the normal para-neoplastic tissues for the two markers were all negative staining.

**Table 1:** Association between eIF4E expression, integrin αvβ6 expression and clinicopathologic variables in colon cancer cases

**Clinicopathological factors**	**n**	**eIF4E expression**		**Value**	**Integrin αvβ6 expression**		**P Value**
		**High (n = 74)**	**Low (n = 64)**		**Positive (n = 54)**	**Negative (n = 84)**	
Gender				0.345			0.927
Male	76	38	38		30	46	
Female	62	36	26		24	38	
Age(years)				0.874			0.108
<60	70	38	32		32	38	
≥60	68	36	32		22	46	
Tumor anatomical location				0.634			0.773
Right hemicolon cancer	72	32	40		43	29	
Left hemicolon cancer	66	32	34		41	25	
T stage				0.072			0.011
T1^#^	1	0	1		0	1	
T2^#^	13	8	5		2	11	
T3	55	23	32		17	38	
T4	69	43	26		35	34	
N stage				0.067			0.109
N0	76	34	42		26	50	
N1	44	28	16		17	27	
N2	18	12	6		11	7	
M stage							
M0	114	57	57	0.063	40	74	0.034
M1	24	17	7		14	10	
TNM stage				0.009			0.012
I - II	72	31	41		21	51	
III-IV	66	43	23		33	33	
Differentiation				0.959			0.209
Well	50	26	24		15	35	
Moderate	55	30	25		23	32	
Poor/undifferentiated	33	18	15		16	17	
Survival (60-month follow-up)				0.013*			0.025*
Death	63	40	23		30	33	
Censored	75	34	41		24	51	

Footnote: ^#^Date of T1 and T2 were combined into one set for chi-square test.

*Log-rank test.

### Clinicopathologic data

After our TMA-IHC staining, all 138 cases for eIF4E and integrin αvβ6 were assessable. The detailed clinicopathologic data for eIF4E and integrin αvβ6 are outlined in Table 1.

### Association between eIF4E and clinicopathologic variables and patient survival

There was significant association between eIF4E and TNM stage (P = 0.009). The high eIF4E rate of TNM III-IV specimens was 65.2%, much more than that of TNM I – II 43.1%. According to the standard of P < 0.05,eIF4E expression has no significant association with other clinicopathologic factors, tumor’s anatomical location included, while P-Values of T stage, N stage and M stage on eIF4E were all between 0.05 and 0.10.

**Figure 3:**
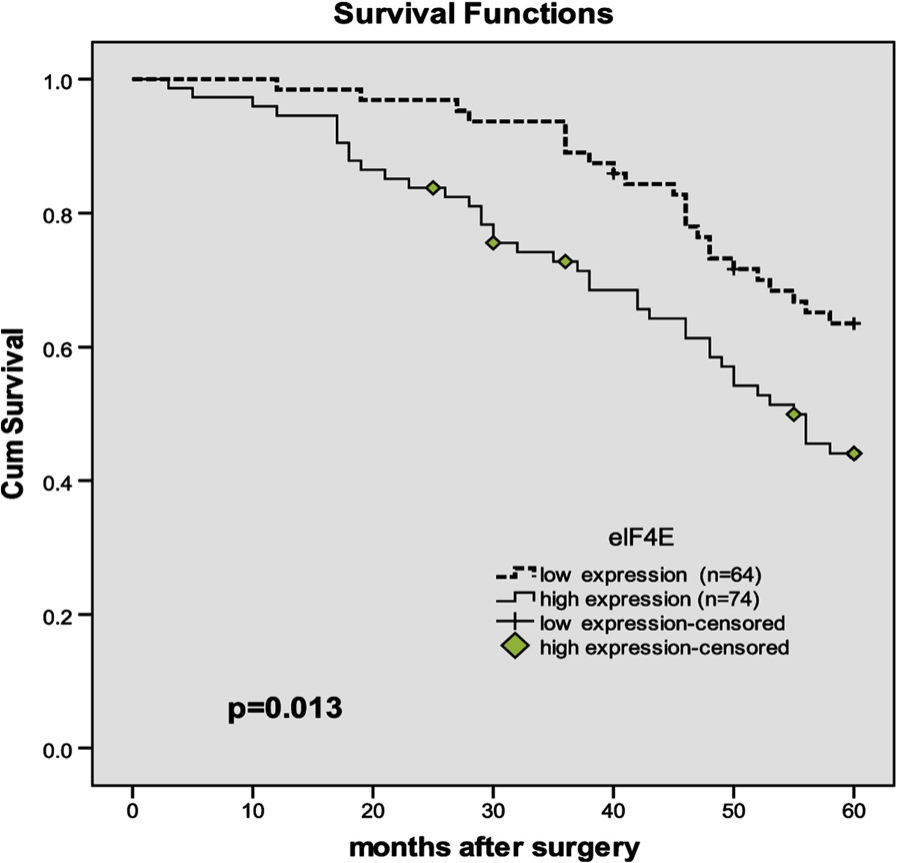
Overall survival according to eIF4E expression (P = 0.013, The log-rank test).

Survival curves were generated through the Kaplan-Meier survival analysis, Patients with high eIF4E expression had a significantly poorer overall survival than those with low expression (P = 0.013, The log-rank test, *χ*^2^ = 6.418). Patient survival over time on eIF4E expression is illustrated in Figure 3.

### Association between integrin αvβ6 and clinicopathologic variables and patient survival

There was significant association between integrin αvβ6 expression and T stage (P = 0.011),TNM stage (P = 0.012). The positive integrin αvβ6 rate of TNM III-IV specimens was 50.0%, much more than that of TNM I – II 29.2%. Based on the standard of P < 0.05,integrin αvβ6 expression has no association with other clinicopathologic factors including age, gender, tumor’s anatomical location, pathology grade, N stage and M stage.

**Figure 4:**
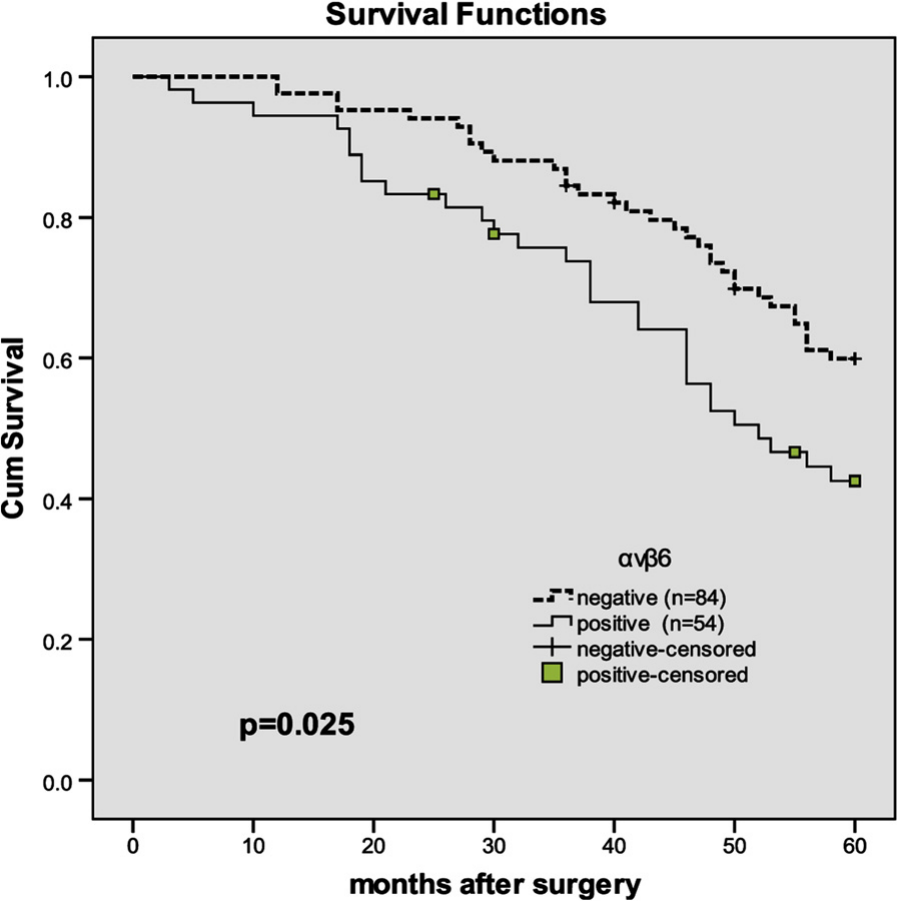
Overall survival according to αvβ6 expression (P = 0.025, The log-rank test).

Patients with high positive αvβ6 expression had a significantly poorer overall survival rate than those with negative expression (P = 0.025, The log-rank test, *χ*^2^ = 5.012). Patient survival over time on eIF4E expression is illustrated in Figure 4.

### The correlation between integrin αvβ6 and eIF4E expression in colon cancer

Positive integrin αvβ6 expression was observed in 52.7% of high eIF4E expression tissue, while the rate in low eIF4E expression tissue was 23.4%. Based on the Spearman correlation analysis, integrin αvβ6 expression had a positive correlation with eIF4E expression (r =0.299, P <0.001, Table 2).

**Table 2:** Correlation between integrin αvβ6 expression and eIF4E expression in human colonic carcinoma tissues (r = 0.299, P < 0.001)

**eIF4E**	**Integrin αvβ6**		**Total**
	**Negative**	**Positive**	
Low	49	15	64
High	35	39	74
Total	84	54	138

**Figure 5:**
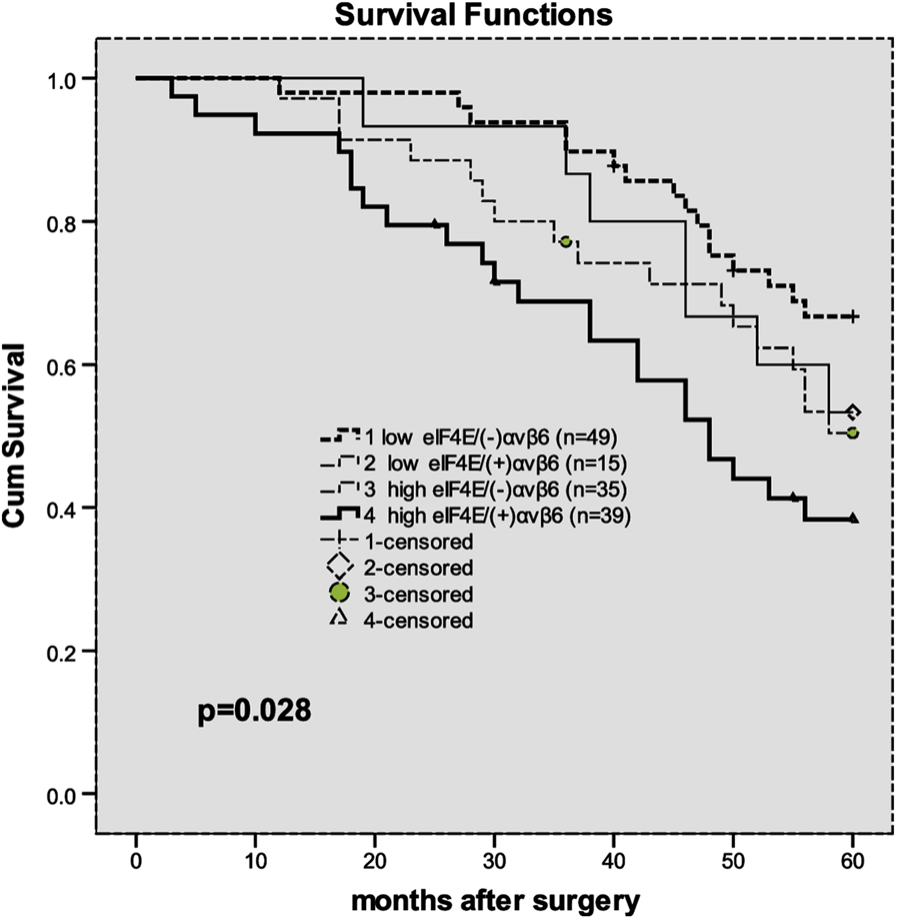
Overall survival according to eIF4E with integrin αvβ6 expression (P = 0.028, The log-rank test).

These 138 cases were stratified according to eIF4E and integrin αvβ6 expression into 4 groups: group 1, low eIF4E/(−) integrin αvβ6 (n = 49); group 2, low eIF4E/(+) integrin αvβ6 (n =15); group 3, high eIF4E/(−) integrin αvβ6 (n =35); and group 4, high eIF4E/(+) integrin αvβ6 (n = 39). Patients with high eIF4E/(+) integrin αvβ6 had a significantly poorer overall survival rate than other groups (P = 0.028, The log-rank test, *χ*^2^ = 9.091). Patient survival over time on eIF4E with integrin αvβ6 expression is illustrated in Figure 5.

### Univariate and multivariate analysis for prognosis of patients with colon cancer

Univariate and multivariate data analyses were performed using the Cox proportional hazards regression model to determine the prognostic value of eIF4E and integrin αvβ6 expression. Older age at diagnosis, high eIF4E expression and positive expression of αvβ6 were the factors to predict a poor prognosis in univariate analysis (P < 0.001,0.016, 0.028,respectively) (Shown in Table 3). Then the variables with P < 0.10 were chosen to conduct multivariate analysis, we revealed that high eIF4E expression and positive expression of αvβ6 were unfavorable independent prognostic factors (relative risk (RR): 2.417 and 2.393; P = 0.001 and 0.001,respectively), while increased age, poor/undifferentiated pathology grade were also independent prognostic factors (RR: 1.114 and 2.195; P < 0.001 and 0.003, respectively) (Shown in Table 3).

**Table 3:** Univariate and multivariate analysis of association of clinicopathologic features with 5-year survival in colon cancer cases

**Variable**	**Univariate analysis**			**Multivariate analysis**		
	**Relative risk**	**95% CI**	**P Value**	**Relative risk**	**95% CI**	**P Value**
Age at diagnosis	1.091	1.063, 1.119	<0.001	1.114	1.083, 1.145	<0.001
Gender						
Male	1.000(Ref.)	-	-			
Female	0.729	0.438, 1.212	0.223			
Tumor anatomical location						
Right hemicolon cancer	1.000(Ref.)	-	-			
Left hemicolon cancer	1.000	0.610,1.640	0.999			
T stage						
T1	0.000	0.000, >10^5^	0.973			
T2	0.951	0.395, 2.290	0.910			
T3	1.152	0.685, 1.937	0.595			
T4	1.000(Ref.)	-	-			
N stage						
N0	1.000(Ref.)	-	-			
N1	1.308	0.760, 2.252	0.332			
N2	1.356	0.646, 2.843	0.421			
M stage						
M0	1.000(Ref.)	-	-			
M1	1.308	0.697, 2.454	0.403			
TNM stage						
I	1.000(Ref.)	-	-			
II	0.634	0.242, 1.664	0.355			
III	0.998	0.378, 2.638	0.997			
IV	1.029	0.362, 2.922	0.957			
Pathology Grade						
Well	1.000(Ref.)	-	-	1.000(Ref.)	-	-
Moderate	1.666	0.912, 3.046	0.097	1.348	0.721, 2.519	0.349
Poor/undifferentiated	1.845	0.950, 3.583	0.070	2.915	1.453, 5.848	0.003
eIF4E						
Low expression	1.000(Ref.)	-	-	1.000(Ref.)	-	-
High expression	1.885	1.128, 3.150	0.016	2.417	1.413, 4.134	0.001
αvβ6						
Negative	1.000(Ref.)	-	-	1.000(Ref.)	-	-
Positive	1.741	1.061, 2.857	0.028	2.393	1.421, 4.028	0.001

## Discussion

Colorectal cancer has a higher morbidity and mortality in developed countries [19]. Despite improvements made in screening and treatments during the past years, the clinical outcome of colon cancer remains unsatisfactory. Understanding the molecular mechanism of tumorigenesis would contribute to treatment of patients with this disease.

Over the past decades, the contribution of the mRNA cap-binding protein, eIF-4E, to malignant transformation and progression has been illuminated. eIF4E gene is reported to be recognized as an proto-oncogene *in vivo*,and the extent of eIF4E overexpression can predict cancer recurrence and outcome in head neck squamous cell carcinoma (HNSCC), breast cancer and lung cancer [9,20]. In our present study, patients with high expression eIF4E tended to have a poorer prognosis than those with low expression. And the level of eIF4E expression had significant association with TNM stage, while P-Values of T stage, N stage and M stage respectively on eIF4E were all between 0.05 and 0.10. From this point, we could speculate that, to some extent, the expression of eIF4E was related to tumor’s metastasis and progression. In brief, the association between eIF4E expression and poor prognosis could be explained by the hypothesis of “strong and weak mRNAs”. Translation of strong mRNAs (e.g. those encoding housekeeping proteins such as beta-actin) will quickly reach a maximum in the presence of low levels of free eIF4E while weak mRNAs are poorly translated. With the increasing number of free activated eIF4E, the translation of these “weak mRNAs” relating to malignancy is disproportionately enhanced, which contributes to the malignant activities [21,22].

The integrin αvβ6 occurs in many epithelial tumors and has been considered to play a prominent role in tumor invasion and metastasis [14]. Recently, it was reported as an independent unfavorable prognostic indicator in aggressive colonic and gastric carcinomas of humans [12,13], which was confirmed in our study according to the Cox regression model. Similar to the study of Bates [12], our results also showed that the αvβ6 expression were significantly associated with clinical stage and the depth of invasion of tumors. These results could be explained logically by our previous studies, that αvβ6 serves to direct growth factor-activated ERK to downstream cytoplasmic targets involved in regulating cell growth, apoptosis and cytoskeletal reorganization [23,24], and in promoting cellular migration by mediating MMP-9 secretion [25].

Integrin-dependent translational control in cancer progression has been widely investigated and recognized in recent years [16]. The mechanism involves the ability of integrins to activate the PI-3 K/Akt/mTOR and Ras-ERK-MNK pathway, which subsequently enhance eIF4E function through the inhibition of 4E-BP1 [16-18,26] and phosphorylation of eIF4E at ser209 by MNK [27]. Released eIF4E, accompanied with eIF4G and eIF4A, is then able to engage and activate eIF4F complex, leading to translation initiation of genes associated with malignancy. The role of integrin on translation has been addressed in models requiring Integrin β3, Integrin β4, and Integrin β1 (reviewed in [16]).

In this study, we revealed that high expression of eIF4E combined with positive αvβ6 can predict a poorer prognosis. And their expressions were positively correlated in colon cancer. However, integrin αvβ6 dependent translational control hasn’t been investigated before. As a member of integrin families, integrin αvβ6 could trigger ERK pathway, leading to the activation of downstream signals [23,24]. According to the theoretical basis mentioned above, we can logically speculate that integrin αvβ6 probably could also mediate the activation of eIF4E, leading to translation of “weak mRNA” consequently. On the other side, another bold but reasonable hypothesis is that the activated eIF4E could up-regulate the protein synthesis of Integrin β6, as the mRNA of Integrin β6 has long 5′-UTRs with rich GC, complex structure (Gene ID: 3694, from NCBI), meeting the criteria of “weak mRNAs”. Thus, a signal loop involving eIF4E andαvβ6 probably exists to play a key role in tumor cellular growth and migration. Of course, our further work is to demonstrate our hypotheses and determine whether it can be translated into real clinical benefit.

## Conclusion

In summary, our findings indicated that the levels of eIF4E and αvβ6 expression are elevated in colonic carcinoma, which were associated with tumor progression and poor prognosis of patients with colon cancer. Additionally, the expression of eIF4E and integrin αvβ6 are moderate-poorly correlated, and with cautious speculation, they may interact with each other at molecular levels, with significant implications for therapeutic intervention.

## Materials and methods

### Clinical samples

Our study was a retrospective analysis of patients who had been diagnosed as colon cancer at Qilu Hospital of Shandong University, between January 2006 and July 2008. Included in this study, patients with colon cancer must have received surgical resection as the initial treatment modality without major perioperative complications, had adequate archived tissue kept and complete clinicopathologic data obtained . This resulted in a collection of tissue from 138 patients, 76 males and 62 females with a median age of 57.4 years and an age range of 22–86 years. Based on tumor’s anatomical location, we classified colon cancer into right hemicolon cancer (including cecum, ascending colon, and right transverse colon) and left hemicolon cancer (including left transverse colon and descending colon). Among those, 63 (45.7%) were confirmed cancer-specific death within 5 years of prognosis and 75 (54.3%) were censored as their case follow up was discontinued or patients were alive beyond 60 months or died of reasons other than colon cancer. The pathologic tumor–node–metastasis (TNM) classification was based on the criteria of the International Union Against Cancer (2009). The study complied with the requirements of The Ethics Committee of Qilu Hospital, Shandong University. And the detailed case characteristics are summarized in Table 1.

### Antibodies and TMA-immunohistochemistry

As primary antibodies, prediluted anti-Integrin β6 polyclonal rabbit antibody (1:200; Proteintech Group, Inc., Cat.No.19695-1-AP, Chicago, USA), anti- eIF-4E rabbit monoclonal antibody (1:100; Epitomics, Inc., Cat.No. 1598–1, Burlingame, USA) were used. HRP secondary antibodies and DAB kit were obtained from ZSGB-BIO (Beijing ZSJQB Co., Ltd., Beijing, China).

First, the 138 patients’ paraffin blocks were collected from the archive of Pathology Department of Qilu Hospitital. Then colonic tumor tissue microarrays (TMA) were constructed as described previously [28], and two successive 5 μm-thick TMA sections were cut ready for immunohistochemistry. There were 150 spaced, array pattern cores (1 mm in diameter) on either section containing 138 carcinoma specimen and 12 paracancerous normal tissue.

Next, immunohistochemical staining for the two markers was done on TMA sections as follows. Sections were incubated for 60 min at 65°C, deparafinized and rehydrated using a xylene and ethanol series. Then microwave antigen retrieval was performed: sections were spaced in 250 mL citrate buffer (pH 6.4) for microwaving, with high-temperature for 5 minutes and 40°C for 15 minutes. After cooled to room temperature and washed in PBS, the tissue was quenched of endogenous peroxidase by 3% H_2_O_2_ for 20 minutes, and blocked in goat serum 37°C for 40 minutes. Primary antibody incubation was then done by either antibody respectively overnight at 4°C. The second day, TMA tissue was incubated with universal IgG antibody-Fab-HRP polymer for 30 min; then, DAB and Hematoxylin were stained and terminated in time sequentially. Finally, the samples were observed under light microscope (Olympus Corp, Tokyo, Japan) after sealed with neutral tree gum on slides.

### Evaluation of and integrin αvβ6 immunohistochemistry

integrin αvβ6 was expressed both in cytoplasm and on cellular membrane, mainly seen on the internal surface of the tumor epithelial cell membrane [29]. The expression of eIF4E was mainly observed in cytoplasm of tumor cells. Semi-quantitative expression levels were evaluated by three individuals based on the average intensity and percentage of positively stained tumor cells. Staining intensity was graded as follows: 0 (no staining), 1 (weak staining = light yellow), 2 (moderate staining = yellow brown), and 3 (strong staining = brown). And the percentage of stained tumor cells was scored as 0 (no positive cells), 1 (less than 25% positive cells), 2 (25–50% positive cells), 3 (more than 50-75% positive cells), and 4 (more than 75% positive cells). Consensus results were determined for cores with different initial scores, and ultimate scoring was validated by a consultant histopathologist. An intensity score ≥2 with at least 50% of tumor cells with positive eIF4E staining was used to classify tumor patients with high expression group,and the others including negative staining were considered as low expression group. In the tumor, positive αvβ6 staining was judged by the presence of an unequivocal brown staining in the cytoplasm of 10% or more of tumor cells, the rest were negative.

### Statistical analysis

Statistical analyses were performed with SPSS 13.0 software. A chi-square test of cross-tabulations and a Fisher exact test were used to examine the relationship between the expression of eIF4E and integrin αvβ6 and their clinicopathologic characteristics. Survival analyses were conducted by the Kaplan-Meier method and the log-rank test. A Spearman correlation was applied to evaluate the relationship between eIF4E and integrin αvβ6 expression levels. Both univariate and Multivariate analysis for cancer specific deaths were done with the Cox proportional hazard model. P-values < 0.05 was considered to be statistically significant.

## Abbreviations

eIF4E: eukaryotic initiation factor 4E; VEGF: Vascular endothelial growth factor; FGF-2: Fibroblast growth factor; MMP-9: Matrix metalloprotease 9; ECM: Extracellular matrix; TMA: Tissue microarrays; 4E-BP: 4E-binding protein; PI-3 K: Phosphatidylinositol-3 kinase; mTOR: mammalian target of rapamycin; ERK: Extracellular regulated protein kinase.

## Competing interests

The authors declare that they have no any competing interests.

## Authors’ contributions

NJ was responsible for designing of the study and critical review of manuscript; NZ and WJ were responsible for designing and performing of the study, literature research and manuscript writing; MS, SQ, LB were responsible for data acquisition; NW, LE, PC, OS were responsible for data analysis; MS, HZ, LS, ZX were responsible for critical review of manuscript. All authors approved the final version of the manuscript.
